# Paradoxical role of CBX8 in proliferation and metastasis of colorectal cancer

**DOI:** 10.18632/oncotarget.2502

**Published:** 2014-10-24

**Authors:** Jianjun Tang, Gang Wang, Meifang Zhang, Feng-yan Li, Yi Sang, Boqing Wang, Kaishun Hu, Yuanzhong Wu, Rongzhen Luo, Dan Liao, Jingying Cao, Xin Wang, Li Wang, Ruhua Zhang, Xiaoshi Zhang, Wu-Guo Deng, Dan Xie, Rui-hua Xu, Tiebang Kang

**Affiliations:** ^1^ State Key Laboratory of Oncology in South China, Collaborative Innovation Center for Cancer Medicine, Sun Yat-Sen University Cancer Center, Guangzhou 510060, China; ^2^ Department of Hepatobiliarypancreatic Surgery, Affiliated Tumor Hospital, Xinjiang Medical University, Urumqi 830000, China

**Keywords:** CBX8, Polycomb Group protein, colorectal cancer, metastasis, proliferation, p53, integrin

## Abstract

The effect of polycomb chromobox (Cbx) proteins in cancer is context-dependent. The Chromobox homolog 8 (CBX8) was originally characterized as a transcriptional repressor, which inhibits cell proliferation in Ink4a-Arf-dependent and -independent manner. However, the role of CBX8 in colorectal cancer remains unknown. Here, we found that high CBX8 expression was associated with a low rate of distant metastasis and good prognosis in CRC patients, even though CBX8 was up-regulated in CRC cell lines and clinical samples. Knockdown of CBX8 inhibited CRC proliferation in vitro and in vivo, mostly by increasing p53 and its downstream effectors. However, knockdown of CBX8 enhanced CRC migration, invasion and metastasis in vitro and in vivo, in part through direct up-regulation of integrin β4 (ITGB4) that in turn decreased RhoA activity. Collectively, the knockdown of CBX8 inhibited CRC proliferation, while promoting its metastasis, thus exerting paradoxical effects in CRC progression.

## INTRODUCTION

Colorectal carcinoma (CRC) is among the four leading causes of cancer-related deaths. Despite the progress made in diagnosis and therapeutic strategy, CRC patients often develop local recurrence and metastasis to the lymph nodes, liver, and lungs at late stages, which dramatically decrease the 5-year-survival rates from nearly 90% to 19%. Therefore, it is necessary to investigate the mechanisms of carcinogenesis and CRC progression and to find potential biomarkers to improve CRC diagnosis, prognosis and treatment prediction [1–6].

The Polycomb Group (PcG) protein family is a class of proteins that epigenetically repress gene expression through chromatin histone modifications [7, 8] and plays important roles in stem cell differentiation and in tumor carcinogenesis and metastasis [9, 10]. The polycomb chromobox (Cbx) proteins, including five members (CBX2, CBX4, CBX6, CBX7 and CBX8) that are homologs of the *Drosophila* Polycomb (Pc) protein, share highly conserved chromodomains and Pc boxes, but their different sizes and the presence of other motifs suggest potentially different functions [11]. Indeed, Cbx proteins confer distinct target selectivity to the Polycomb repressive complex 1 (PRC1) that achieves different functions, such as a balance between the self-renewal and differentiation of embryonic stem cells [12, 13]. However, the roles of Cbx proteins in cancer may be context-dependent and involve other protein complexes [14, 15]. For instance, CBX4 acts as a SUMO E3 ligase and participates in regulating cell senescence [13], transcriptional regulation of proliferation genes [16], DNA damage and repair [17] and tumor angiogenesis and metastasis [18]. CBX7 is a tumor suppressor in multiple cancer types, such as lung cancer, pancreatic cancer, colon cancer and thyroid cancer [19], but serves as an oncogene in gastric cancer and lymphoma [15], [20].

CBX8, also known as HPC3 (Human Polycomb 3), was originally characterized as a transcriptional repressor, interacting with RING1a/b and associating with BMI1 in the PRC1 [21]. It has been reported that as a PRC1 component, CBX8 represses INK4a/ARF expression in fibroblasts [13]. Additional studies showed that several distinct PRC1 complexes colocalize and regulate INK4a/ARF expression, suggesting that the INK4a/ARF locus is a general target for PRC1 complexes rather than a CBX8-specific downstream target [22]. Therefore, the exact role of CBX8 in transcriptional regulation remains largely undefined. It has been reported that certain Cbx proteins, such as CBX4 and CBX8, can associate with protein complexes other than PRC1, thereby playing a PRC1-independent role in transcriptional regulation [11, 13]. However, whether CBX8 has functional roles in CRC remains unknown. In the present report, we found that CBX8 is up-regulated in CRC and is essential for CRC proliferation by suppressing p53, but the knockdown of CBX8 promotes CRC metastasis, most likely by up-regulating integrin β4(ITGB4).

## RESULTS

### CBX8 was up-regulated in human CRC tumor tissues, and the CBX8 knockdown inhibited CRC cell proliferation *in vitro* and *in vivo*

**Figure 1:**
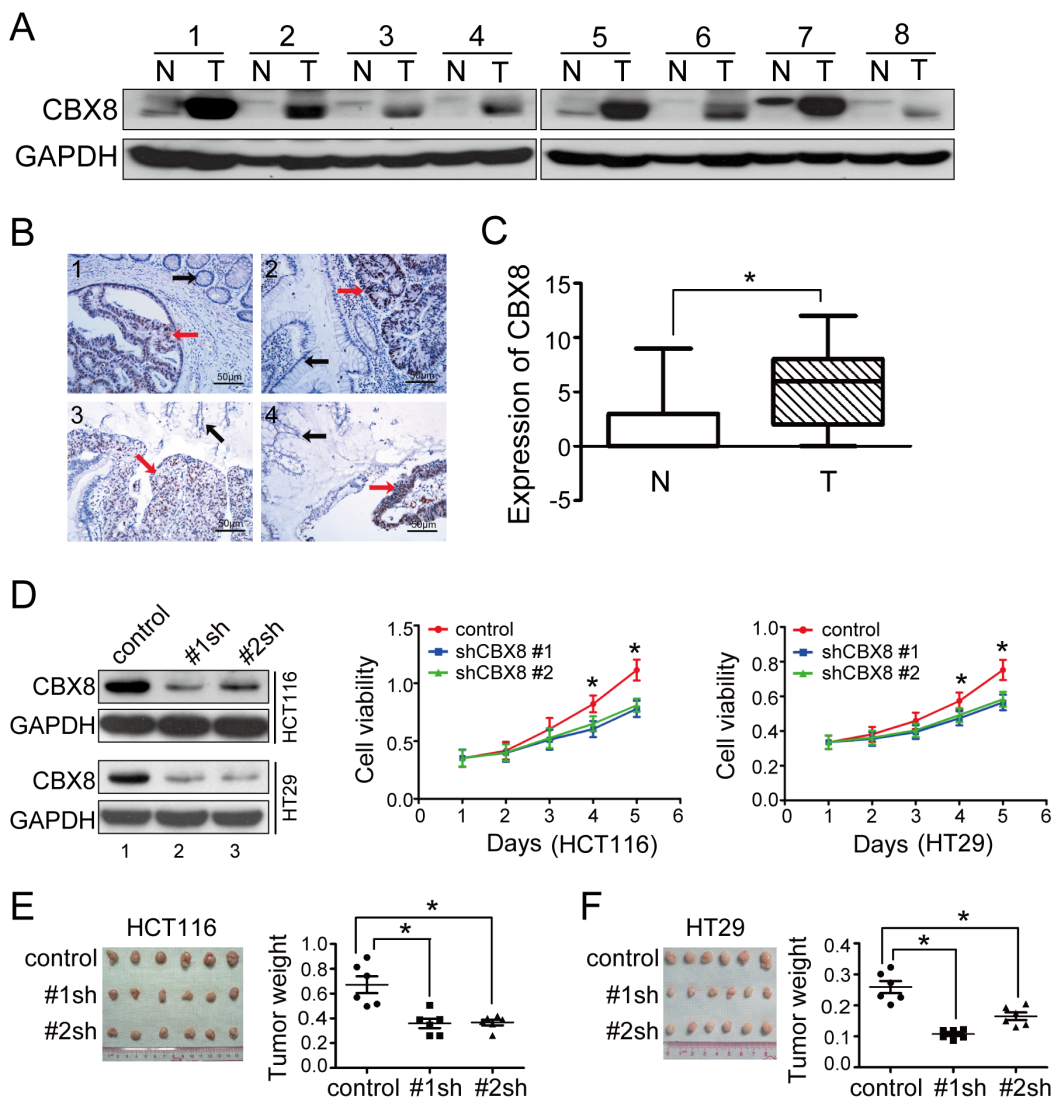
CBX8 was up-regulated and essential for growth in human colorectal cancer (**A**) The CBX8 protein levels in eight pairs of CRC tumor tissues (T) and their non-tumor counterparts (N) were detected by western blotting. (**B, C**) IHC for CBX8 was performed using 164 clinical CRC samples as described in the Materials and Methods section. Subpanel **B** shows 4 representative images, and subpanel **C** illustrates the statistical results of a Mann-Whitney test (*p < 0.05). The dots represent the scores, and the bars indicate the SD. (**D**) In the indicated cell lines, proteins were analyzed (left panel), and cell viability was measured by MTT (middle and right panels). The dots represent the means, and the bars indicate the SD. *p < 0.05 using the independent Student t test (n=3). (**E, F**) The *in vivo* growth of the indicated stable cell lines was examined as described in the Materials and Methods. The images and weight of xenograft tumors are shown in the left and right sides of E and F, respectively. The dots represent the weights, and the bars indicate the SD. *p < 0.05 using the independent Student t test (n=6).

To explore the potential roles of CBX8 in CRC, we collected several paired fresh tissues, including CRC tumor specimens and the corresponding adjacent non-tumor tissue specimens, and RT-PCR and western blot were performed to measure CBX8 mRNA and protein levels in these tissues. As shown in Fig. 1A and Supplementary Fig. S1A, compared with their non-tumor counterparts, CBX8 mRNA and protein levels were significantly elevated in CRC tumor tissues. This up-regulation of CBX8 in CRC was further supported by the IHC results using 164 cases of paraffin-embedded primary CRC specimens (Fig. 1B, 1C), indicating that CBX8 may play an important role in CRC.

In order to determine the functions of CBX8 in CRC, we generated stable transfectants of two specific shRNAs targeting CBX8 in two CRC cell lines, HCT116 and HT-29 (Fig. 1D), because both cell lines have higher CBX8 protein levels compared with other CRC cell lines and CCD-18-Co, a transformed colonic cell line (Supplementary Fig. S1B). Using the MTT assay and as shown in Fig. 1D, the cell viabilities of both HCT116 and HT29 obviously decreased when CBX8 was knocked down. More importantly, as shown in Fig. 1E, the CRC xenograft tumor growth of these stable transfectants was clearly impaired *in vivo* when CBX8 was knocked down in both HCT116 and HT29 cells. Altogether, we concluded that CBX8 may play an essential role in CRC proliferation.

In contrast, the cell viabilities of HCT116 and HT29 cells stably expressing ectopic CBX8 were marginally altered (Supplementary Fig. S2A, S2B). Furthermore, using DLD1 cell line, which has lower CBX8 protein level compared with other CRC cell lines (Supplementary Fig. S1B), the cell viability was not changed when CBX8 was stably overexpressed in this cell line in vitro, and the CRC xenograft tumor growth of this stable transfectant was not impaired neither in vivo (Supplementary Fig. S2C, S2D, S2E). These results indicate that either endogenous CBX8 is enough to execute these observed functions or that ectopic CBX8 needs other components to function properly in HCT116 and HT29 cells.

### The inhibitory effect of CBX8 knockdown on CRC cell proliferation was mostly p53-dependent

**Figure 2:**
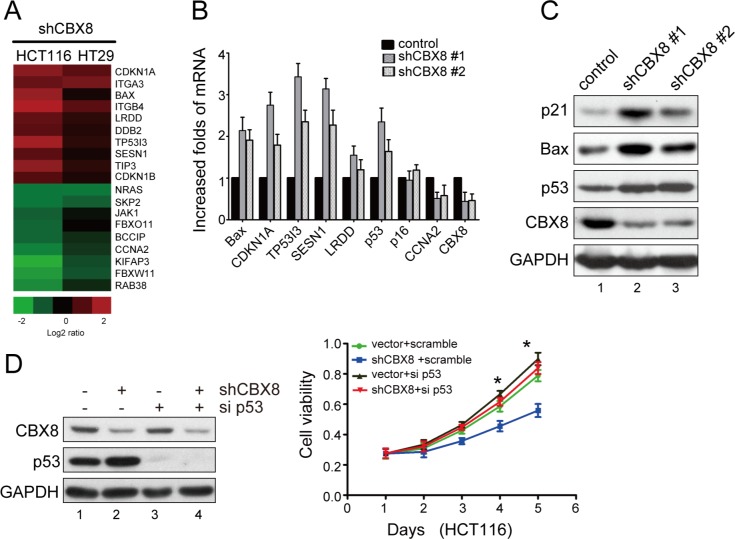

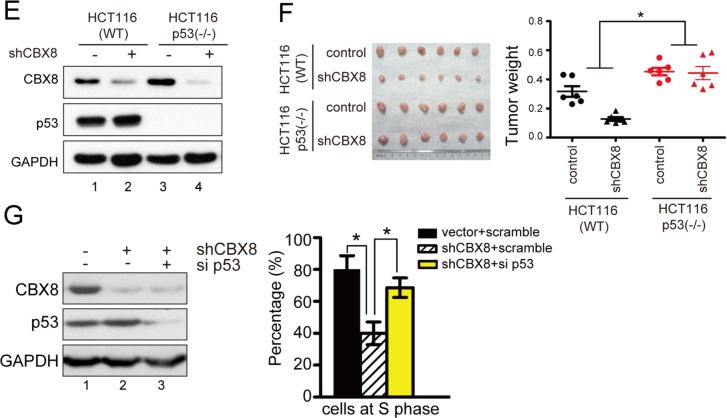
The cell proliferation inhibition after CBX8 knockdown was mostly dependent on p53 (**A**) Gene expression profiles with the stable knockdown of CBX8 in HCT116 and HT29 cells as indicated. (**B**) The relative mRNA levels of the indicated genes normalized to GAPDH levels in the indicated cell lines were determined by qRT-PCR (mean ± SD of triplicate samples are shown). (**C**) The proteins in the indicated cell lines were analyzed by Western blotting. (**D**) Cell viability and the proteins in HCT116 cells treated as indicated were analyzed by MTT (right panel) and Western blotting (left panel), respectively. The dots represent the means, and the bars indicate the SD. *p < 0.05 using independent Student t test (n=3). (**E, F)** The *in vivo* growth of the indicated stable cell lines was examined as described in the Materials and Methods. The proteins in the indicated cells were analyzed by Western blotting (**E**). The images and weight of xenograft tumors are shown in the left and right side of **F**, respectively. The dots represent the weights, and the bars indicate the SD. *p < 0.05 using the independent Student t test (n=6). (**G**) HCT116 cells under the indicated conditions were synchronized at G1/S boundary by double thymidine block and released for 3 hours to proceed into S phase, as described in the Materials and Methods. The proteins in the indicated conditions were analyzed by Western blotting (left panel), and the percentages of cells at S phase are shown (right panel). The results are expressed as the mean ± SD of three independent experiments, *p < 0.05.

Because it has been reported that CBX8 affects the proliferation of fibroblasts by directly suppressing the INK4A-ARF locus [23], we tested if this effect was present in CRC cells. Unfortunately, the knockdown of CBX8 did not affect the expression of p16ink4a-arf in HCT116 and HT29 cells (data not shown), indicating that CBX8 inhibits the growth of CRC cells independent of p16^ink4a-arf^. To investigate the inhibitory mechanism of CBX8 in CRC growth, we utilized a whole genome microarray with stable shRNA-CBX8 transfectants in HCT116 and HT-29 cells (Fig. 1D). In general, because the Cbx proteins are considered transcriptional suppressors [11], we focused on the genes up-regulated after CBX8 knockdown. As shown in Fig. 2A, several p53 targets, such as BAX, CDKN1A (p21), TP53I3, SESN3 and LRDD, were mostly increased, but p16 remained constant when CBX8 was knocked down. These results were further validated by qRT-PCR (Fig. 2B), indicating that the p53 pathway may be involved in the CBX8-mediated proliferation of CRC cells. Indeed, as shown in Fig. 2C, the protein levels of p53 and its downstream effectors, including BAX and CDKN1A (p21), were significantly increased in the shRNA-CBX8 cells. Furthermore, as shown in Fig. 2D and Supplementary Fig. S3, the reduction in cell viability caused by CBX8 knockdown was mostly rescued when p53 was simultaneously knocked down in HCT116 and HT29 cells, demonstrating that the inhibitory effect of CBX knockdown on cell proliferation is mostly p53-dependent in CRC cells. More importantly, using both HCT116-WT and HCT116-p53^−/−^ cell lines, as shown in Fig. 2E, 2F, the CRC xenograft tumor growth was clearly impaired in HCT116-WT cells, but not in HCT116-p53^−/−^ cells, in vivo when CBX8 was stably knocked down in these cells, reinforcing the notion that the inhibition of CBX8 knockdown on cell growth mainly relies on p53 in CRC cells.

Based on the fact that the p53/p21 axis plays a key role in cell cycle procession and particularly in the G1/S transition, HCT116 cells were synchronized at the G1/S boundary and released into S phase. As shown in Fig. 2G, the number of cells released from the G1/S boundary into S phase was obviously reduced in the shRNA-CBX8 cells compared with the control cells. This effect was mostly rescued by a simultaneously knockdown of p53, indicating that the inhibition of CBX8 knockdown on CRC proliferation is mainly due to the p53-induced impairment of cell cycle progression.

### Low levels of CBX8 were associated with a poor prognosis in CRC patients

**Figure 3:**
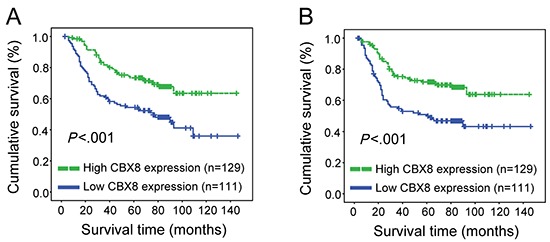
CBX8 expression correlated with overall survival and disease-free survival (**A, B**) Overall survival (**A**) and disease-free survival (**B**) curves were generated based on the CBX8 protein expression statuses from 240 paraffin-embedded CRC tumor tissues. Actuarial probabilities were calculated using the Kaplan-Meier method and were compared using the log-rank test. The patients with high CBX8 expression in their primary tumors had a better overall survival (**A**) and disease-free survival (**B**) rates compared with low CBX8 expressing patients.

Next, we sought to investigate the clinical significance of CBX8 in CRC, and 240 paraffin-embedded primary CRC specimens were collected and examined by IHC using an anti-CBX8 antibody (Supplementary Fig. S4). As shown in Supplementary Table 1, CBX8 expression levels closely correlated with several clinicopathological characteristics in CRC patients, such as clinical stage (p = 0.004), T classification (p = 0.011), M classification (p = 0.003). Furthermore, multivariate Cox regression analysis revealed that the CBX8 expression levels, N classification and M classification were independent prognostic factors for CRC patients (Supplementary Table 2). Using Kaplan-Meier analysis and to our surprise, low expression levels of CBX8 were associated with a poor disease-free survival and overall survival in CRC patients (Fig. 3). These results were completely unexpected. We hypothesized that high but not low CBX8 expression would indicate a high clinical stage and high rate of distant metastasis because higher CBX8 levels were detected in CRC tumor tissues compared with their non-tumor counterparts, indicating that CBX8 may also be critical for metastasis along with its role in CRC proliferation.

### CBX8 knockdown promoted CRC migration, invasion and metastasis

**Figure 4:**
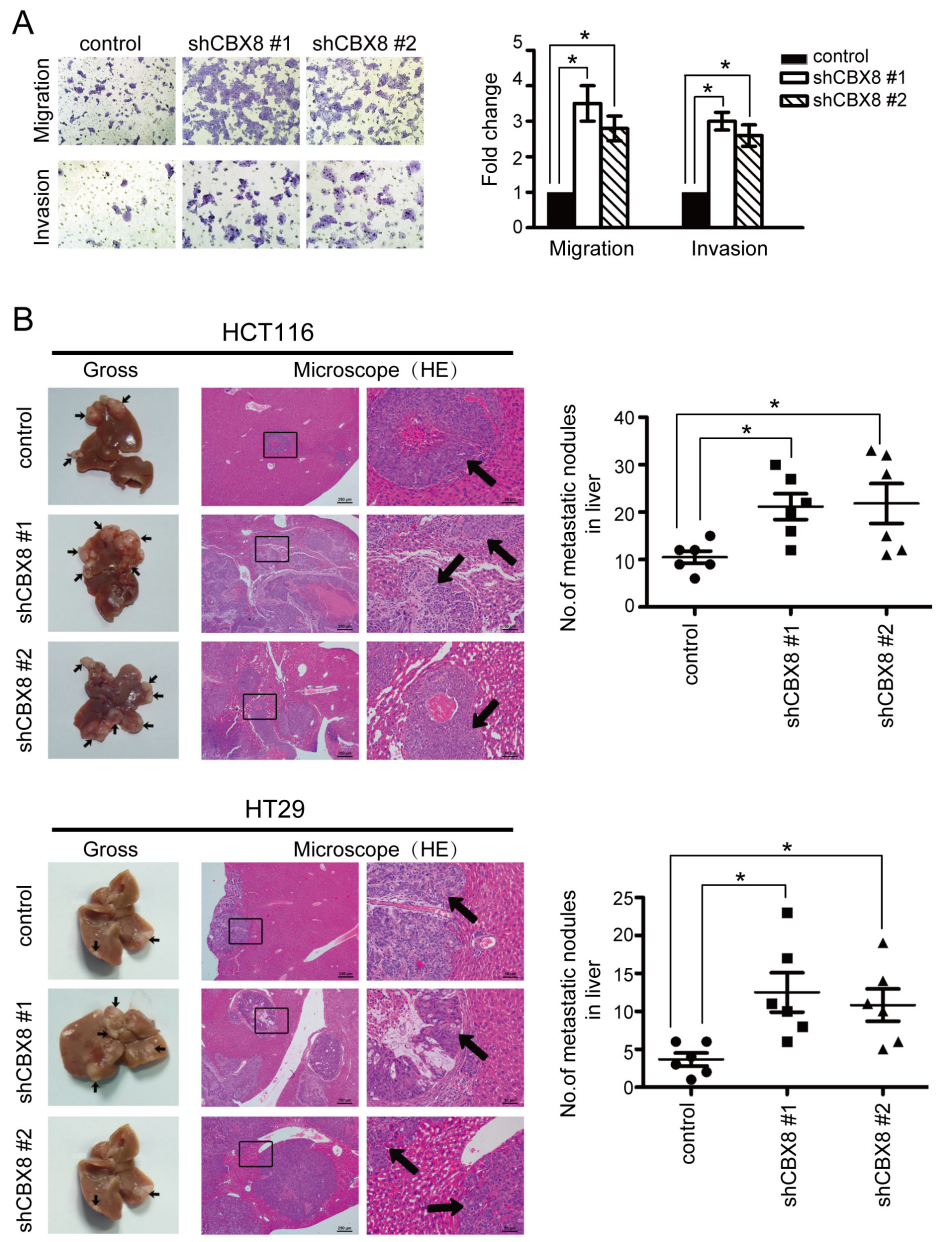
The knockdown of CBX8 enhanced CRC cell motility and metastasis (**A**) HCT116 cell migration and invasion were determined as described in the Materials and Methods (n=3). The bars indicate the SD. *p < 0.05 using Student's *t*-test. (**B**) *In vivo* liver metastasis of the indicated stable cell lines in nude mice was determined as described in the Materials and Methods. The left panels present representative results of gross and H&E staining of metastatic liver nodules. Arrows indicate the metastatic nodules. The right panels illustrate the statistical results (n=6). The bars indicate the SD. * *p* < 0.05 using Student's *t*-test.

The unexpected above-described results (Fig. 3) prompted us to re-analyze the whole genome profiles after CBX8 knockdown using KEGG Pathway analysis. As shown in Supplementary Fig. S5, the pathways related to metastasis, such as the regulation of actin cytoskeleton, focal adhesions and tight junctions, were also significantly altered when CBX8 was knocked down. We sought to test if motility was also altered in the stable shRNA-CBX8 transfectants in HCT116 cells (Fig. 1D). The migration and invasion capabilities of HCT116 cells were dramatically increased by knocking down CBX8 (Fig. 4A). Likewise, the cell migration and invasion abilities were not changed when HCT116 cells were stably overexpressed of ectopic CBX8 (Supplementary Fig. S6), consistent with the notion that either endogenous CBX8 is enough to execute its functions or that ectopic CBX8 needs other components to function properly in HCT116 cells. More importantly, using the hepatic metastasis *in vivo* animal model, the sizes and numbers of metastatic nodules were significantly increased after CBX8 knockdown in both HCT116 and HT-29 cells (Fig. 4B). These results demonstrated that the knockdown of CBX8 promotes CRC migration, invasion and metastasis.

### The ITGB4/RhoA axis may be critical in the enhancement of CRC metastasis induced by CBX8 knockdown

**Figure 5:**
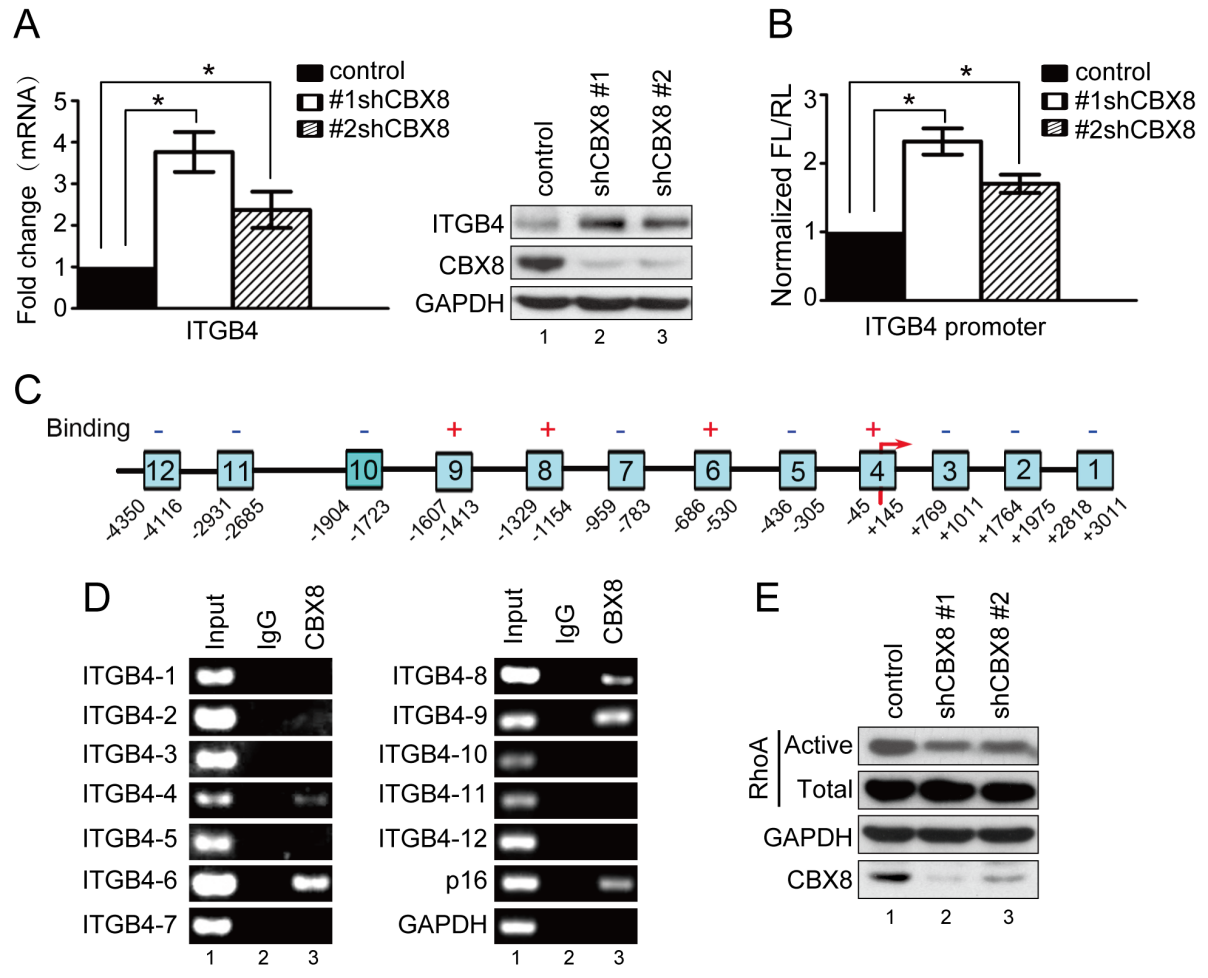
The knockdown of CBX8 increased ITGB4 expression and down-regulated RhoA activity (**A**) The relative mRNA levels of ITGB4 were normalized to the GAPDH levels in the indicated cell lines, as assessed by qRT-PCR (left panel, mean ± SD of triplicate samples are shown). The proteins in the indicated cell lines were analyzed by Western blotting (right panel). (**B**) The relative luciferase activity of ITGB4 in the indicted cells was determined as in the Materials and Methods section. (**C**) A schematic illustration of the ITGB4 promoter regions (1–12) with (+) or without (−) binding affinity for CBX8. The arrow indicates the transcriptional start site. (**D**) A ChIP assay was performed with HCT116 cells using an anti-CBX8 antibody or IgG antibody as indicated. The p16 and GAPDH promoters were used as the positive and negative controls, respectively. (**E**) Western blotting was performed after the pull-down of activated forms of RhoA in the indicated HCT116 cells as described in the Materials and Methods section.

Next, we sought to determine the target involved in CBX8-mediated motility in CRC, and the up-regulation of ITGB4 after CBX8 knockdown caught our attention (Fig. 2A), as ITGB4 is a key component for both actin cytoskeleton rearrangements and focal adhesions and plays a critical role in cell motility [24–28]. Indeed, increasing ITGB4 by shRNA-CBX8 was further validated by qRT-PCR and western blot (Fig. 5A). Interestingly, the knockdown of CBX8 increased ITGB4 promoter activity measured by the luciferase reporter assay (Fig. 5B). Furthermore, using chromatin immunoprecipitation (ChIP) and as shown in Fig. 5C and 5D, CBX8 was clearly associated with several regions of the ITGB4 promoter. Collectively, we conclude that CBX8 associates with and suppresses the ITGB4 promoter, which in turn impairs ITGB4 expression. These data were further supported by phalloidin staining, which showed the cortical localization of F-actin and lamellipodia morphology in shCBX8-HT29 cells (Supplementary Fig. S7). Given that Rho GTPases, including RhoA, Rac1 and Cdc42, are key players in cell motility and are closely related to ITGB4 [29–35], a GTPase activity pull-down assay was performed to measure Rho GTPase activity in the CBX8-knockdown CRC cells. As shown in Fig. 5E, active RhoA was obviously decreased when CBX8 was knocked down, but the knockdown of CBX8 did not affect the activity of Rac 1 nor Cdc42 (Supplementary Fig. S8). Taken together, these results indicated that the knockdown of CBX8 removes its inhibition on the ITGB4 promoter to increase ITGB4 protein expression, which reduces active RhoA and then results in actin rearrangements that enhance CRC metastasis.

## DISCUSSION

**Figure 6:**
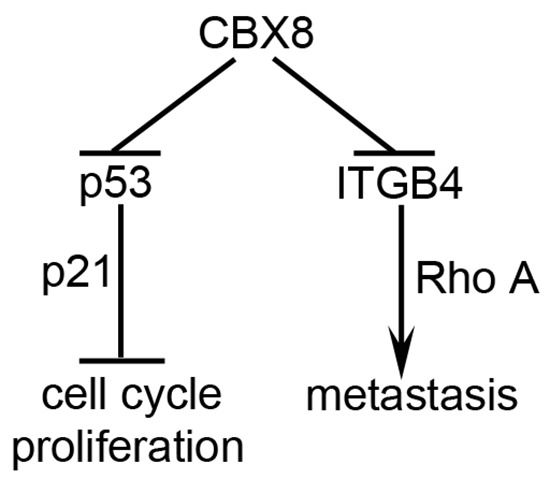
A proposed model for the functions of CBX8 in CRC proliferation and metastasis In CRC, CBX8 may mainly suppress p53 and its downstream p21, and consequently plays an essential role in the cell cycle progression and cell proliferation, in the meanwhile, CBX8 may also directly associate with and repress ITGB4 promoter, which modulates RhoA activity and then metastasis.

CRC is a multiple-stage and multiple-step continuous process from early carcinogenesis to advanced stages with distant metastasis. During this process, multiple genes, such as K-RAS, APC, p53 and BRAF, are genetic and epigenetic altered [2]. In this report, CBX8 up-regulation was detected in CRC cell lines and clinical samples and contributed to CRC proliferation by suppressing p53. However, the knockdown of CBX8 promoted CRC metastasis, likely through the ITGB4/RhoA axis, as illustrated in Fig. 6. The paradoxical functions of CBX8 in CRC progression may provide a new insight into CRC.

It has been reported that CBX8 regulates proliferation directly by transcriptional suppression of p16Ink4a/Arf in a PRC1-dependent or -independent manner [23]. Here, we found that CBX8 may repress p53, but not p16Ink4a/Arf, to promote proliferation in CRC cells (Fig. 2). This finding is consistent with the notion of Ink4a-Arf-dependent and -independent functions of CBX8 in cell proliferation [23], and may also be important during the CRC progression where p53 is a key player [23], as CBX8 may be another layer to regulate p53 in CRC. In addition, an interaction between CBX8 and p53 was detected in CRC cells, as shown in Supplementary Fig. S9. Therefore, we reason that p53 acetylation related to either TIP60 or SIRT1 may be crucial for this CBX8-induced inhibition of p53 because TIP60 often acts as a functional partner for CBX8 [23, 36–38], and CBX8 suppresses the stress-induced premature senescence of human breast cancer cells through cooperation with SIRT1 to suppress p53 acetylation [39]. Moreover, how CBX8 suppresses p53 in CRC needs to be determined, as CBX8 was undetectable in the p53 promoter region using HCT116 cells (Supplementary Fig. S10). CBX8 could have different mechanisms in different cancer types, as CBX7 also plays diverse roles in different cancers [14, 15, 19, 40]. Furthermore, EZH2 has oncogenic activity in multiple cancers, but inactivating mutations lead to the tumor suppressor activity of EZH2 in myeloid malignancies [41–43].

Strikingly, CBX8 negatively regulates motility while promoting CRC proliferation, as the knockdown of CBX8 inhibited proliferation but enhanced CRC metastasis (Fig. 1, 2, 4). The ITGB4/RhoA axis may be critical for this CBX8 function in CRC metastasis. First, the direct binding of CBX8 to the ITGB4 promoter was detected by ChIP. Second, the knockdown of CBX8 enhanced ITGB4 promoter activity, increased ITGB4 expression and decreased active RhoA, which leads to the rearrangement of the actin cytoskeleton that enhances cell motility [32–35]. These paradoxical functions in CRC proliferation and metastasis for CBX8 imply that CBX8 may play essential role in early stages of CRC when proliferation is dominant and p53 is functional, while CBX8 may inhibit cancer cell invasion and metastasis at late stages of CRC when functions of p53 were lost [23]. We believe that CBX8 is a tumor suppressor in CRC considering that CRC patients with low CBX8 expression had high rate of distant metastasis (Fig. 3). However, CBX8 does act as an oncogene in the transcriptional regulation and leukemogenesis of MLL-AF9 [23, 38].

Recently, the paradoxical functions in proliferation and metastasis for certain molecules have emerged. For example, high c-Myc suppresses cancer metastasis, most likely through the direct transcriptional repression of integrin subunits [44], and cyclin A2 negatively controls cell motility by promoting RhoA activation and cytoskeletal rearrangements [32]. These new data showed that both c-Myc and cyclin A2, two well-established proliferation markers, were able to inhibit cancer metastasis [44]. All of these mechanisms for both c-Myc and cyclin A2, such as integrin, RhoA and cytoskeletal rearrangements, seem to be involved in CBX8 inhibition in CRC metastasis. Therefore, CBX8, c-Myc and cyclin A2 are most likely not good therapeutic targets; however, they are able to promote proliferation even while their inhibition may enhance metastasis, indicating that current strategies mainly targeting proliferation may have undesirable effects on tumor progression.

## MATERIALS AND METHODS

### Human tissue specimens and cell lines

A total of 240 paraffin-embedded primary specimens were obtained from the recruited CRC patients. The patients were diagnosed according to their clinicopathological characteristics at the Sun Yat-Sen University Cancer Center, Guangzhou, China, from 1999 to 2007. No patients had received radiotherapy and/or chemotherapy prior to surgery. Eight CRC tissue specimens and the corresponding adjacent non-tumor tissue specimens were stored at –80°C immediately after surgery and were used for quantitative RT-PCR and Western blotting. Informed consent was obtained from all patients and approved by the Institutional Research Ethics Committee. The clinicopathological characteristics of these CRC patients were shown in Supplementary Table 1.

Four colorectal tumor cell lines (HT-29, HCT116, SW620 and DLD-1), an embryonic kidney cell line 293T and a normal colonic cell line CCD-18-Co were obtained from the American Type Culture Collection. Cells were cultured and stored according to the instructions of American Type Culture Collection.

### MTT assay

3-(4,5-Dimethylthiazol-2-yl)-2,5-diphenyltetrazolium bromide (MTT) assay was used to measure the cell viability. Briefly, the shRNA-treated HCT116 and HT29 cells were seeded at a density of 2500 and 3000 cells per well in 96-well microplates, respectively. The cells were incubated with MTT for 4 hours, the optical density (OD) was detected at 490 nm with a microplate reader, and measurements were acquired once per day for 5 days. The results were presented as the mean ± SD of three independent experiments.

### Cell synchronization and FACS analysis

Cells were seeded on 6-well plates at 30% confluence and synchronized at the G1/S boundary by double thymidine. These procedures were previously described [45]. Briefly, cells were treated with 2 mM thymidine for 16 hours, released in fresh medium containing 10% fetal bovine serum (FBS) for 9 hours and incubated with 2 mM thymidine for another 16 hours. At this point, approximately 90% of the cells were synchronized at G1/S boundary and then released a second time, and cells were collected cells at 0 and 3 hour time points. The cell cycle profiles were analyzed by flow cytometry (FACS), which was performed as previously described [45].

### Western blotting and immunoprecipitation

Western blotting was performed as previously described [45, 46]. Briefly, cells were lysed in MCLB (50 mM Tris-HCl [pH 8.0], 2 mM DTT, 5 mM EDTA, 0.5 % Nonidet P-40, 100 mM NaCl, 1 μM microcystin, 1 mM sodium orthovanadate, 2 μM PMSF, protease (Sigma Chemical Co.) and phosphatase inhibitor cocktail [Calbiochem]), and the clarified lysates were resolved by SDS-PAGE and transferred to PVDF membranes for western blotting using ECL detection reagents (Beyotime Co.). For immunoprecipitation (IP), the clarified supernatants were first incubated with anti-FLAG-agarose (Sigma Aldrich) or anti-HA-agarose (Sigma Aldrich) gels for 2 hours to overnight at 4°C, and the precipitates were washed five times with MCLB. To investigate the interaction between endogenous CBX8 and p53, the clarified supernatants were first incubated with an anti-CBX8 antibody (Bethyl Laboratories, Inc.) for 2 hours at 4°C. Protein A/G-agarose was then added for 2 hours to overnight, and the precipitates were washed five times with MCLB and analyzed by western blotting.

### In vitro migration and invasion assays

Transwell chambers (BD Biosciences) were used for the migration and invasion assay. One hundred microliters of a 2×10^6^/ml-cell suspension in serum-free medium were added to the upper chamber, which was coated with (invasion assay) or without (migration assay) a matrigel mix, and six hundred microliters of medium 20 % FBS-containing medium were placed into the lower chamber as a chemoattractant. After 48 (invasion assay) or 24 hours (migration assay), cells on the lower side of the upper chamber were fixed with methanol, stained with 0.1 % crystal violet, and air dried. The number of invading or migrating cells was determined with a microscope by counting five different fields.

### In vivo tumor growth and metastasis assays

Female BABL/c athymic nude mice (4 weeks old) were obtained from the Animal Center of Guangdong province (Guangzhou, China). All the animal experiments were performed according to the National Institutes of Health Animal Use Guidelines on the Use of Experimental Animals. To assess in vivo CRC tumor growth, 2×10^6^ cells were injected subcutaneously into the dorsal flanks of each mouse. Each group included six mice. Tumor size was measured every 3 days, and the tumor volume was estimated. After 4 weeks, the mice were euthanized, and the tumors were removed and weighed.

For a hepatic metastasis model, the mice were subjected to laparotomy. The spleen was accessed through a 1-cm incision in the upper left lateral abdomen, and 2×10^6^ cells suspended in 20 μl of phosphate-buffered saline were injected into the distal tip of the spleen using an insulin syringe. The spleen was then replaced in the abdomen, and the abdominal cavity was closed with sutures. After 6 weeks, the mice were euthanized, and the spleens and livers were removed for pathological examination. Metastatic nodules were counted in a double-blind manner using microscopy.

### Immunohistochemistry (IHC)

Immunohistochemical staining for CBX8 and IHC evaluation in human CRC tissues was performed as previously described [47]. Briefly, sections of paraffin-embedded specimens were baked and deparaffinized. Sections were stained with an anti-CBX8 antibody (Sigma Aldrich), and the antigen was detected using a secondary anti-rabbit HRP-conjugated antibody and a DAB chromogen kit (Genetech) followed by counterstaining with hematoxylin. CBX8 expression was evaluated by two independent pathologists. The immunohistochemical staining score was calculated according to the percentage of positive tumor cells and the staining intensity.

### Luciferase reporter and chromatin immunoprecipitation (chip) assays

Luciferase reporter gene constructs containing the ITGB4 promoter were cloned into the promoter-less pGL3 enhancer plasmid vector. Luciferase activity was measured using the Dual-Luciferase Reporter Assay System (Promega) as previously described [46]. Cells at 50% confluence in 12-well dishes were transfected using the Lipofectamine® 2000 reagent (Life Technologies). All experiments were performed in triplicate. The expression levels of firefly and renilla luciferases were analyzed 48 hours after transfection according to the manufacturer's instructions.

ChIP was performed using the EZ-Magna ChIP™ A/G One-Day Chromatin Immunoprecipitation Kits (Millipore). Briefly, adherent mammalian cells at ~80–90 % confluence in a 150-mm culture dish were treated with formaldehyde to cross-link chromatin-associated proteins to DNA. According to the manufacturer's instructions, cells were lysed and sonicated to generate DNA fragments of 200–1000 bp (confirmed by agarose gel electrophoresis). Then, equal aliquots of sonicated chromatin samples were subjected to IP overnight using anti-CBX8 antibodies or anti-IgG as a negative control. DNA was purified to remove the chromatin proteins, and the ITGB4 promoter was amplified by qRT-PCR. All assays were performed three to four times, and representative results were shown.

### GTPase activity pull-down assays

This assay was performed with the RhoA/Rac1/Cdc42 Activation Assay Combo BiochemKit (Cytoskeleton, Cat. # BK030). Briefly, cells were lysed in buffer containing protease inhibitors and incubated for 1 hour at 4°C with either 50 μg rhotekin-Rho-binding domain protein GST-beads for RhoA or 10 μg p21-activated kinase 1(PAK)-p21-binding domain protein GST beads for Rac1 and Cdc42. Samples were subjected to electrophoresis on a 12% SDS-PAGE gel. Then, proteins were transferred to PVDF, probed with anti-RhoA (CST, Cat. #2117), anti-Rac1 (CST, Cat. #2465) and anti-Cdc42 (BD, 610929) antibodies, and processed for chemiluminescent detection. The amount of activated Rho was determined by measuring band intensities.

### Statistical analysis

The SPSS software (version 16.0, SPSS Inc., Chicago, IL, USA) was used for statistical analysis. Statistical significance was tested by Student's t-test or a chi-square test, as appropriate. The relationship between CBX8 expression and the clinicopathological parameters was examined by the Pearson chi-square and Kruskal-Wallis tests. The correlations between CBX8 expression and overall and disease-free survival curves were plotted by the Kaplan-Meier method and compared with the log rank test. Univariate and multivariate Cox regression analyses were used to evaluate survival data. Differences were considered statistically significant when p values were < 0.05.

## SUPPLEMENTARY METHODS TABLES AND FIGURES


