# Supplementary material for: Paradoxical role of CBX8 in proliferation and metastasis of colorectal cancer
Source: Oncotarget. 2014 Oct 24;5(21):10778–90. doi: 10.18632/oncotarget.2502 (PMC4279409; doi:10.18632/oncotarget.2502)
Supplement: Supplementary file 1 [file oncotarget-05-10778-s001.pdf]

SUPPLEMENTARY METHODS TABLES AND FIGURES

RNAi sequence

The pLKO1-CBX8 short hairpin RNA (shRNA) constructs were purchased from sigma addrich corporation. The following CBX8-specific RNAi target sequences into pLKO1: CBX8 shRNA #1: 5-GCGTGAGCTTGGCATAGTGAT-3; CBX8 shRNA #2: 5-GCATGGAATACCTCGTGAAAT-3. Sequences of siRNA target p53 refer to the previous published studies. 5′-GACTCCAGTGGTAATCTAC-3′ (Oncogene. 2011 May 12;30(19):2282–8); 5′-AAGACTCCAGTGGTAATCTAC- 3′ (Mol Biol Cell. 2007;18(7):2630–5);5′-GAAGAAACCACTGGATGGA-3′ (BMC Cancer. 2011 ATg 25;11:378). During the transfection, the three siRNA were mixed as a pool.

Primers for qPCR

Genes	F (5′-3′)	R (5′-3′)	size
CBX8	CCTTCGAAACATGGGTTTGT	CTGGGCTTGTCATCCACTCT	201bp
Bax	CCCGAGAGGTCTTTTTCCGAG	CCAGCCCATGATGGTTCTGAT	155bp
CDKN1A	TGTCCGTCAGAACCCATGC	AAAGTCGAAGTTCCATCGCTC	139bp
p16	GAAGGTCCCTCAGACATCCCC	CCCTGTAGGACCTTCGGTGAC	94bp
TP53I3	GGAGGACCGGAAAACCTCTAC	CCTCAAGTCCCAAAATGTTGCT	166bp
p53	CAGCACATGACGGAGGTTGT	TCATCCAAATACTCCACACGC	125bp
SESN1	TGCTTTGGGCCGTTTGGATAA	TGTAGTGACGATAATGTAGGGGT	131bp
ITGB4	CTCCACCGAGTCAGCCTTC	CGGGTAGTCCTGTGTCCTGTA	133bp
GAPDH	ACAGTCAGCCGCATCTTCTT	GACAAGCTTCCCGTTCTCAG	259bp

Primers for PCR of CHIP assay

ITGB4	F (5′-3′)	R (5′-3′)	size
ITGB4-1	TAAGTGGGCCAGGACAGAAG	TTTGCGAGTTTGTCCCAGTG	194bp
ITGB4-2	CTTCTGTGCCTGGTCTCTCA	GGCATCATTACACAGGCAGG	212bp
ITGB4-3	TTCAGGAAGCTCTACAGGCC	TTCGGACAGCAACAACCATG	243bp
ITGB4-4	TGACTCACCAGCGCCTCCTT	GCCCGTCCTGGACCTACCTCG	190 bp
ITGB4-5	CTGGCACGGAGAGCGGGG	GCCTCTTAGCCCCCGTCC	132bp
ITGB4-6	CATCGAGTGCCAATCCATGCCAG	CCTCCAACCCCTAACCTCACGTTG	157bp
ITGB4-7	TACTCGGGAAACAGGCAGG	CGGGTGTTCAGGTCAGAGAA	177bp
ITGB4-8	GGTGTGGAGAGGTCAAGGAA	TGCCAAACAACACCAGACTG	176bp
ITGB4-9	CCCCTGCAATCACTCTGAGA	GAATCTCGCCACACTGTTGG	195bp
ITGB4-10	TCTCTAATGGGTGCTGCTGG	CCAGTTTGGTTGAAGCACTCA	182bp
ITGB4-11	AGAATCGCTTGAACCCAGGA	TGCTTCCTTCACTCCCAGTT	194bp
ITGB4-12	CTTAGGTGTGAAAAGGGGCC	AGCCTCTGTCTTCTGGGTTC	235bp
p16	GGCATCAGCAAAGTCTGAGC	CTGGGAGACAAGAGCGAAAC	260bp
GAPDH	GGTAGGGAGTTCGAGACCAG	TCAACGCAGTTCAGTTAGGC	290bp

p53	F (5′-3′)	R (5′-3′)	size
p53-1	CGGCAGCCCTGTTATTGTTT	AAATGCAGGCGGAGAATAGC	185 bp
p53-2	TCCTCCCCAACTCCATTTCC	GGACGGTGGCTCTAGACTTT	187 bp
p53-3	GGGAGAAAACGTTAGGGTGTG	TGAGGGCAGAATTGGTGGAA	174 bp
p53-4	GCTGCTGGGAGTTGTAGTCT	TGCCTATATCAGTGCTGGGT	235 bp
p53-5	GAAGCACAGCGGAGATTAGC	GGTGCTAAGGAACACAGTGC	178 bp
p53-6	CACTGCCATCACCACTTACG	GGGACCCAGTTTCTCTCTCC	242 bp
p53-7	GCCCCGTTGTTATCCTTACC	GCGACCCAGGTTTATTGTCC	154 bp
p53-8	GCAGCATTGATGAAGAGGCA	GCCCTAGCCCTACACTTGAA	213 bp
p53-9	AGCTACTTGTGAGGCTGAGG	TCTGCCCCGAATTCTGAAGT	243 bp
p53-10	GCTGCTCTTCTCTGTCCTCA	GCCAAGATCATGTCACTGCA	174 bp
p16	GGCATCAGCAAAGTCTGAGC	CTGGGAGACAAGAGCGAAAC	260 bp
GAPDH	GGTAGGGAGTTCGAGACCAG	TCAACGCAGTTCAGTTAGGC	290 bp

Supplementry Table S1. Correlation between CBX8 expression and clinicopathologic characteristics of colorectal cancer patients

Characteristics	n	CBX8 expression		Chi-square test p value
		Low or none No. cases (%)	High No. cases (%)	
**Gender**				
Male	131	61	70	0.915
Female	109	50	59	
**Age**				
≤50	89	40	49	0.755
>50	151	71	80	
**Clinical Stage**				**0.004***
I+II	95	34	61	
III	90	42	48	
IV	55	35	20	
**T classification**				**0.011***
T1+T2	48	14	48	
T3	66	38	66	
T4	126	59	126	
**N classification**				0.104
N0	113	46	67	
N+	127	65	62	
**M classification**				
M0	185	76	109	**0.003***
M1	55	35	20	
**Pathologic Differentiation**				
Well	23	9	14	0.611
Moderately	160	73	87	
Poorly	57	29	28	
**Histological Types**				0.132
Non-mucinous adenocarcinoma	219	98	121	
Mucinous adenocarcinoma	21	13	8	
**Location**				0.443
colon	121	53	68	
rectal	119	58	61	
**Vital status (at follow-up)**				**0.001***
Alive	144	54	90	
Death	96	57	39	

***p<0.05**

Supplementry Table S2. Univariate and multivariate analysis for overall survival (Cox proportional hazards regression model)

Variable	Univariate analysis		Mutivariate analysis		
	No.	*p* value	HR	95% CI	*p* value
**CBX8 expression**		**<0.001**	0.642	0.420–0.980	**0.041**
low expression	111				
high expression	129				
**Age**		0.461	0.937	0.617–1.423	0.761
≤50	89				
>50	151				
**Gender**		0.818	0.868	0.576–1.308	0.499
Male	131				
Female	109				
**T classification**		**<0.001**	1.206	0.871–1.671	0.259
T1+T2	48				
T3	66				
T4	126				
**N classification**		**<0.001**	2.887	1.781–4.681	**<0.001**
N0	113				
N+ (N1 and N2)	127				
**M classification**		**<0.001**	2.695	1.741–4.173	**<0.001**
M0	185				
M1	55				
**Differentiation**		**0.001**	1.420	0.976–2.067	0.067
Well	23				
Moderately	160				
Poorly	57				

HR: Hazard Ratio; CI, confidence interval; *$p<0.05$

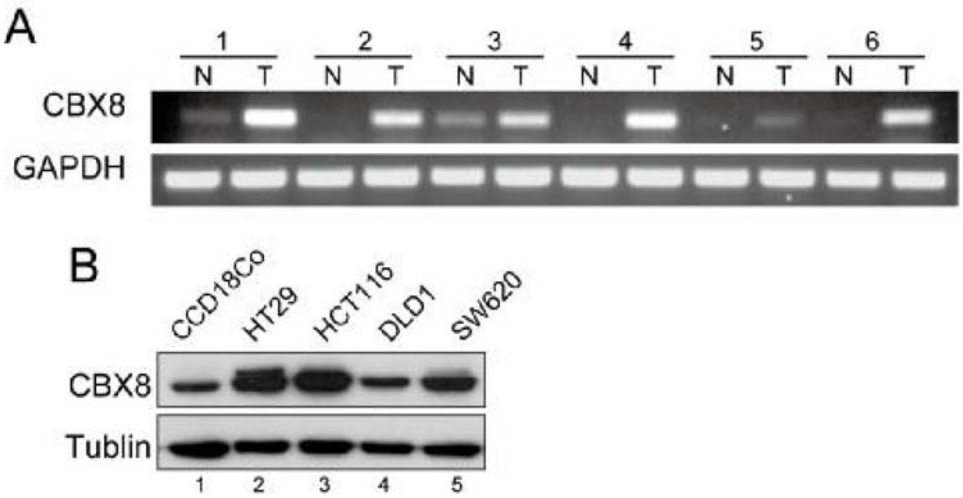

Supplementary Figure S1: CBX8 was up-regulated in human colorectal cancer tissues and cell lines. **(A)** CBX8 mRNA levels in six pairs of CRC tumor tissues (T) and their non-tumor counterparts (N) were assessed by RT-PCR. **(B)** The CBX8 protein levels were visualized in the indicted cell lines by Western Blot.

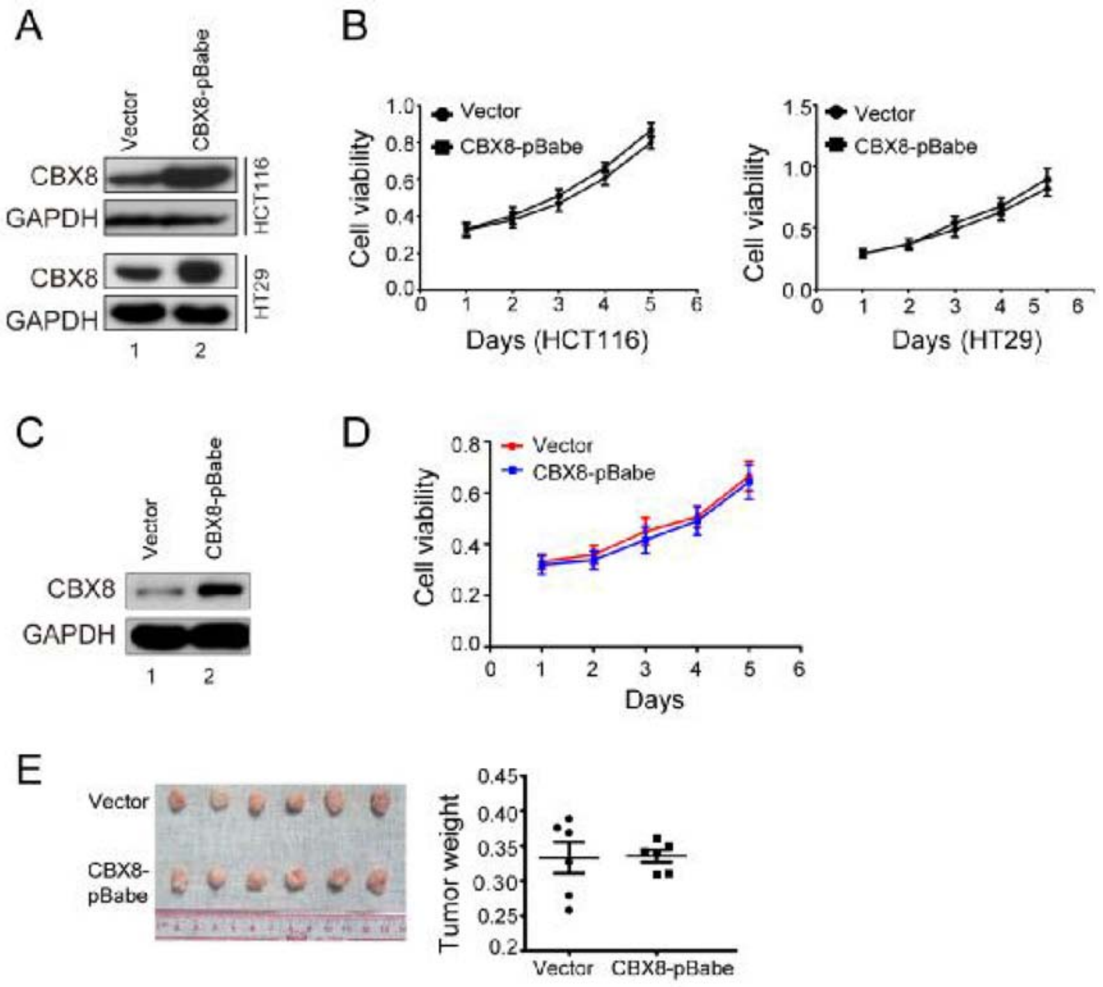

Supplementary Figure S2: Ectopic CBX8 marginally affected CRC cell viability and tumor growth. **(A, B)** HCT116 and HT29 cells stably overexpressing ectopic CBX8 (as indicated) were subjected to Western Blotting **(A)** and MTT assay **(B)** at different time points. The results are expressed as the mean ± SD of three independent experiments. **(C, D)** DLD1 cells stably overexpressing ectopic CBX8 (as indicated) were subjected to Western Blotting **(A)** and MTT assay **(B)** at different time points. The dots represent the means, and the bars indicate the SD. *$p < 0.05$ using the independent Student t test (n=3). The results are expressed as the mean ± SD of three independent experiments. **(E)** The *in vivo* growth of the indicated stable cell lines was examined as described in the Materials and Methods. The images and weight of xenograft tumors are shown in the left and right side, respectively. The dots represent the weights, and the bars indicate the SD. *$p < 0.05$ using the independent Student t test (n=6).

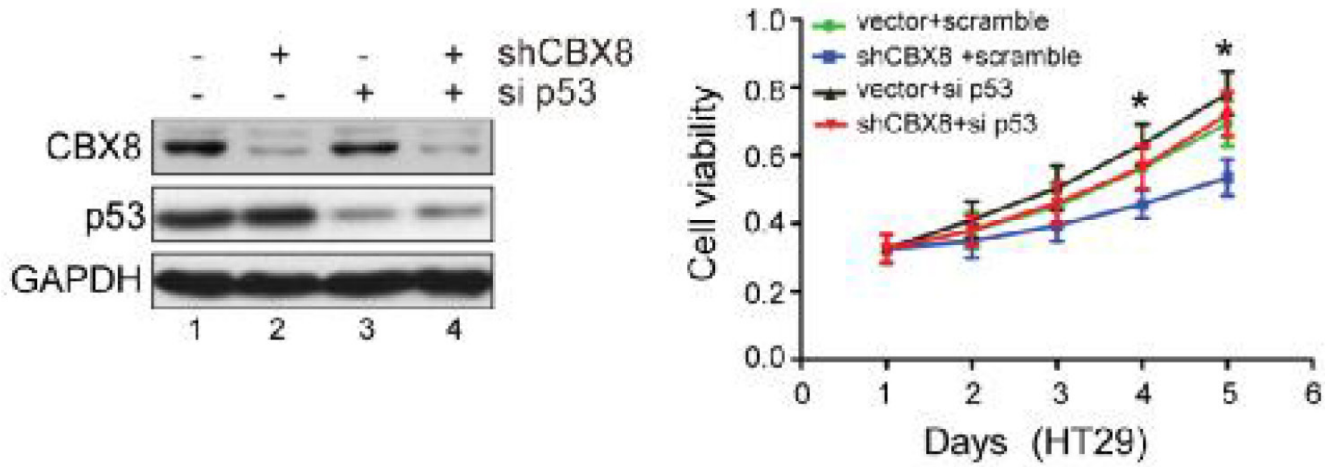

Supplementary Figure S3: The cell proliferation inhibition after CBX8 knockdown was mostly dependent on p53 in HT29 cells. HT29 cells were treated as indicated, proteins were analyzed (left panel) and cell viability was measured by MTT (right panel). The dots represent the means, and the bars indicate the SD. $*p < 0.05$ using the independent Student t test (n=3).

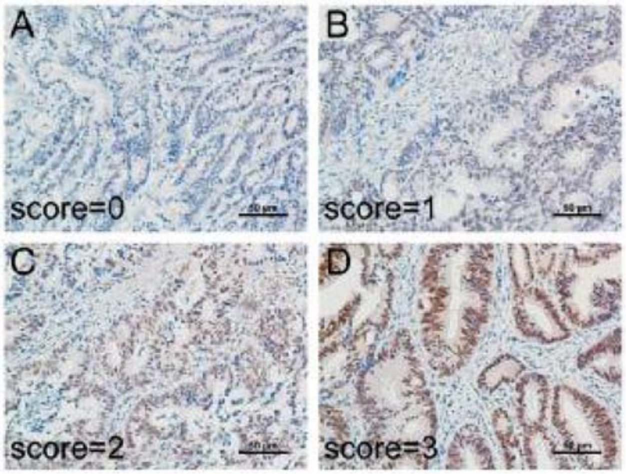

Supplementary Figure S4: IHC grading intensity for CBX8. **(A, B, C, D)** Representative immunohistochemically stained images of CBX8 in CRC. The intensity of the CBX8 signal in CRC was scored as four grades (0, 1, 2, 3), representing negative CBX8 staining **(A)**, weak CBX8 staining **(B)**, moderate CBX8 staining (**C**), and strong CBX8 staining **(D)**, respectively. Image magnification is 200×.

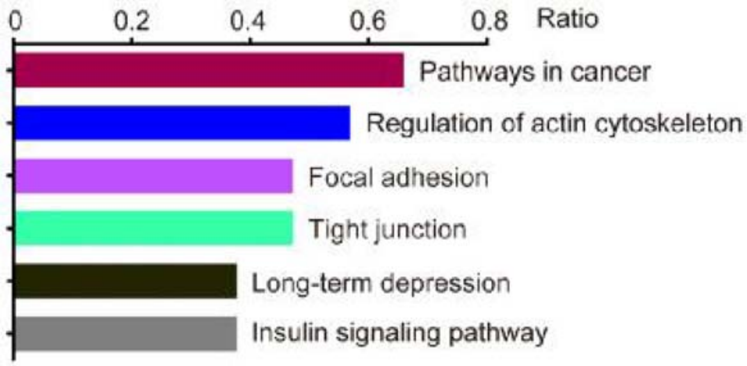

Supplementary Figure S5: Altered pathways in CBX8-knockdown CRC cells. Gene expression profiles were produced in both sh-Control and sh-CBX8 stable transfectants in HT29 and HCT116 cells, and only mRNA levels that changed more than 2-fold were analyzed by KEGG Pathway analysis.

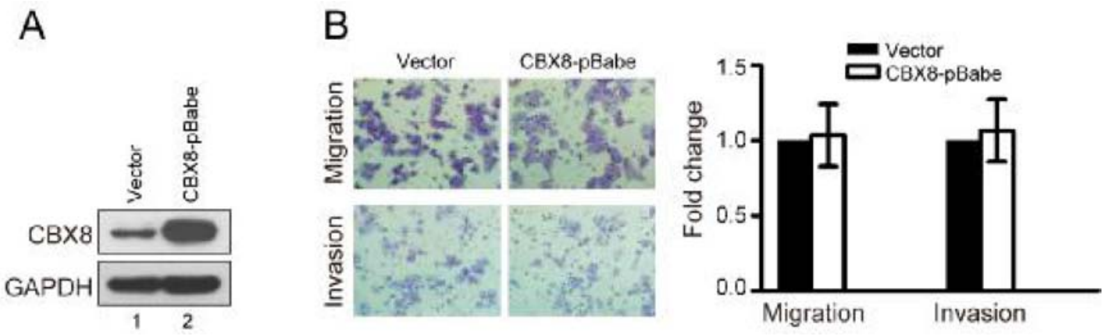

Supplementary Figure S6: Ectopic CBX8 did not change CRC cell motility. HCT116 cells stably vector or overoxpressing CBX8 as indicated were subjected to Western blotting (A), and to cell migration and invasion assay (B) as described in the Materials and Methods (n=3). The results are expressed as the mean ± SD of three independent experiments.

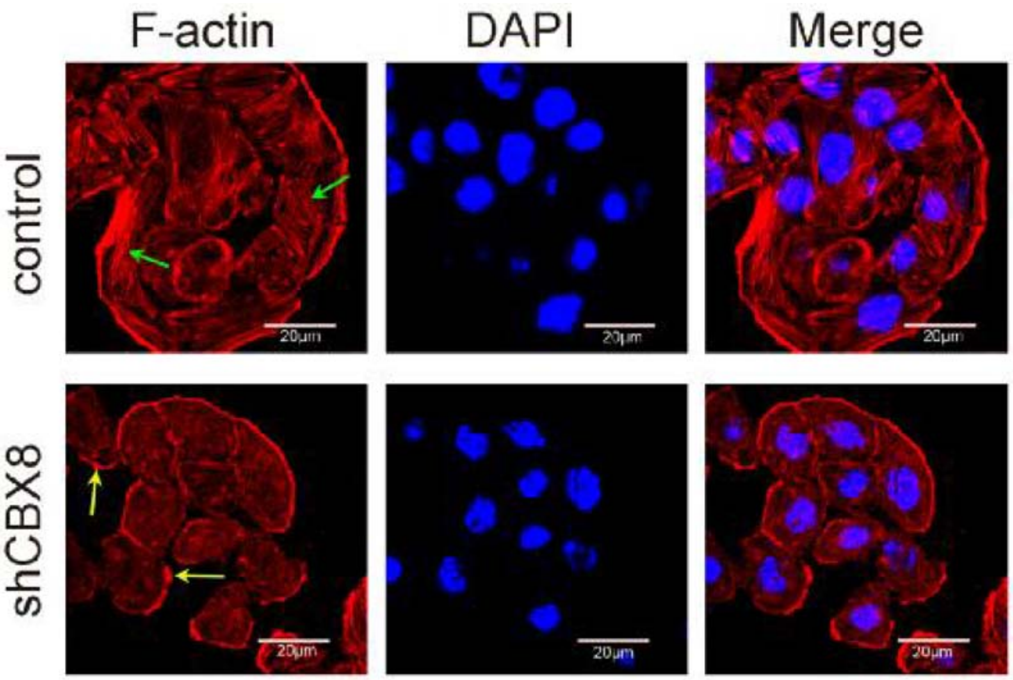

Supplementary Figure S7: The knockdown of CBX8 induced the rearrangement of the actin cytoskeleton. The sh-Control and sh-CBX8 stable HT29 cell transfectants were stained with rhodamine-conjugated phalloidin (a marker of F-actin, red) and DAPI (blue). Scale bars, 20 μm for phase-contrast (green arrow, bundles of actin filaments and yellow arrow, protrusion of cortical actin).

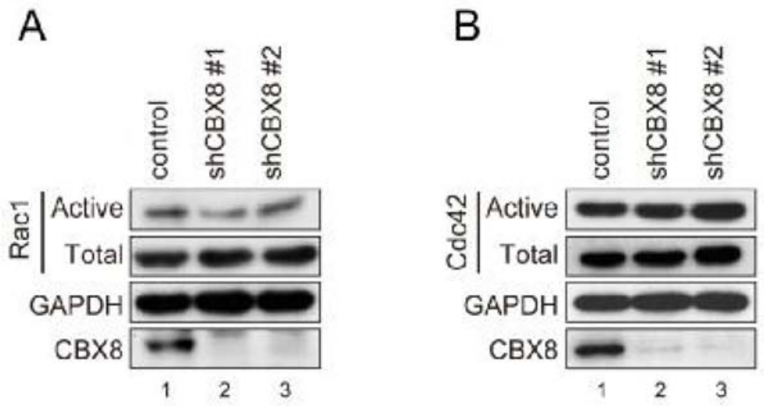

Supplementary Figure S8: The knockdown of CBX8 did not affect the activity of Rac 1 nor Cdc42. **(A, B)** Western blotting was performed after a pull-down of the activated forms of Rac1 and Cdc42 in HCT116 cells, respectively, as described in the Materials and Methods section.

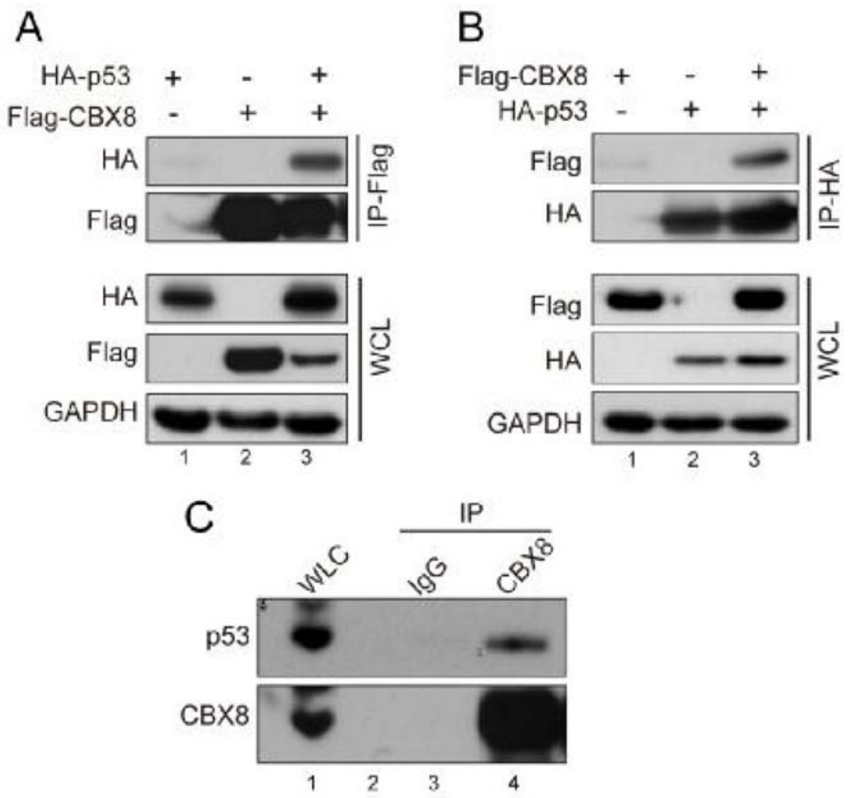

Supplementary Figure S9: CBX8 interacted with p53. **(A, B)** HEK293T cells were transfected with the indicated plasmids for 24 hours and then were lysed with MCLB. IP was performed using anti-Flag antibodies **(A)** or anti-HA antibodies **(B)** and then subjected to Western Blot. **(C)** HCT116 cells were lysed with MCLB. IP was performed using an anti-CBX8 antibody followed by Western Blotting. WCL, whole cell lysate and IP, immunoprecipitation.

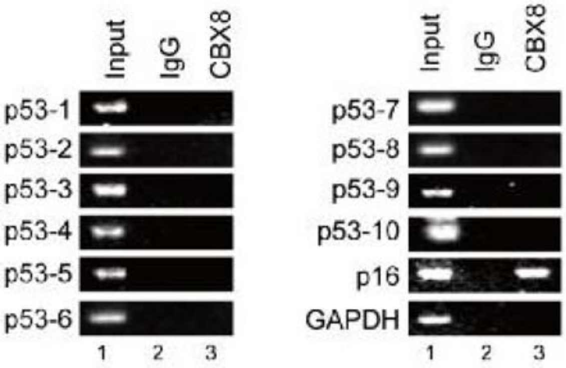

Supplementary Figure S10: CBX8 was undetectable in the p53 promoter region. ChIP assay was performed with HCT116 cells using an anti-CBX8 antibody or IgG antibody as indicated. Ten pairs of primers targeting the p53 promoter and surrounding region were used. The p16 and GAPDH promoters were used as the positive and negative controls, respectively.
